# Estimation of Time Period for Effective Human Inhalational Anthrax Treatment Including Antitoxin Therapy

**DOI:** 10.1371/currents.outbreaks.7896c43f69838f17ce1c2c372e79d55d

**Published:** 2017-07-28

**Authors:** Lewis Rubinson, Alfred Corey, Dan Hanfling

**Affiliations:** University of Maryland School of Medicine, R Adams Cowley Shock Trauma Center, Program in Trauma Critical Care, Baltimore, MD, USA; AC Pharmaco, LLC; Johns Hopkins University, Center for Health Security, Baltimore, Maryland, USA; Department of Emergency Medicine, George Washington University, Washington, DC, USA

## Abstract

**Introduction:**

Infrequent natural human inhalational anthrax cases coupled with high bioterrorism risk have brought about use of animal models to serve as the basis for approval of novel treatments. For inhalational anthrax, protective antigen (PA) drives much of the mortality, and raxibacumab, an anti-PA monoclonal antibody, has been approved for therapeutic use using the Animal Rule. Given the paucity of human inhalational anthrax clinical data including PA kinetics, the post-exposure period for effective treatment of human disease remains unknown. The objective of this investigation was to extrapolate animal PA kinetics to a conceptual human model to estimate the post-exposure period for effective treatment of human inhalational anthrax.

**Methods:**

Human PA kinetic parameters were extrapolated from reported rabbit and monkey data. PA profiles were simulated with and without antibiotic induced PA clearance to represent antibiotic-sensitive and -resistant infections, respectively. Antitoxin levels equimolar to or greater than concurrent PA levels were considered protective.

**Results:**

For antibiotic sensitive infections, treatment with antibiotics alone ≤4 days after spore exposure prevents toxemia. Administration of raxibacumab together with antibiotics protects ≥ 80% of subjects for 3 additional days (7 days post exposure). In the setting of antibiotic resistance, raxibacumab would be protective for at least 6 days post exposure.

**Conclusions:**

Although the animal model of disease does not reflect the potential impact of supportive care (e.g. fluid resuscitation received by critically ill patients) on PA kinetics and raxibacumab PK, the simulations suggest that administration of antitoxin in combination with antibiotics should provide a longer postexposure window for effective treatment than for antibiotics alone. In addition, raxibacumab administration soon after exposure to an antibiotic resistant strain should provide effective treatment.

## Introduction

Inhalational anthrax is caused by the inhalation of *Bacillus anthracis* spores^1^, followed by spore germination and bacterial growth, and is considered a bioterrorism threat^2^^,^^3^^,^^4^^,^^5^^,^^6^. Moderate to large-scale unannounced dissemination of anthrax spores is considered plausible, and due to the complexities and challenges of providing timely post-exposure prophylaxis to all who have been exposed, it is likely that numerous people would develop symptomatic disease. Antibiotics can be used to effectively treat bacteremia, but once patients develop respiratory failure or severe hemodynamic instability, which is largely promoted through toxin-mediated pathogenesis^1^^,^^7^^,^^8^^,^^9^, mortality is very high (up to 45%, despite aggressive critical care and antibiotic administration), even when caused by antibiotic sensitive strains of *B. anthracis*^4^^,^^10^^,^^11^.

The paucity of naturally occurring human clinical cases, especially since the time antitoxin has become available, has made it difficult to derive evidence-based treatment guidelines. Rigorously developed animal models have had to suffice in the absence of human cases, and have allowed for critical evaluation and regulatory approval of novel therapeutics under the Animal Rule, including raxibacumab, an IgG monoclonal antibody against protective antigen (PA)^12^^,^^13^.

PA is largely responsible for the toxin-mediated pathogenesis of inhalational anthrax. The anthrax toxin is a tripartite toxin: lethal factor (LF) and edema factor (EF) have enzymatic activities, while PA binds to cell receptors. PA binds and translocates LF and EF into the cell. Inhibition of PA binding to cell receptors blocks binding and internalization of LF and EF^8^^,^^14^. Administration of raxibacumab has been shown to increase survival in rabbit and monkey models of inhalational anthrax^12^. In rabbits, antibiotics are highly effective when administered at the onset of anthrax disease^15^^,^^16^, but benefit wanes when treatment is delayed until late in disease course. A survival benefit was demonstrated in rabbits when raxibacumab was administered with antibiotics late in the disease course^17^.

Expert panels convened by the US Centers for Disease Control and Prevention have recommended use of approved anthrax antitoxins in combination with antibiotics for treatment of severe anthrax infection including inhalational anthrax^18^^,^^19^. Despite the expert consensus for combination treatment, the effective treatment time period post spore exposure remains unknown. Another publication suggests that patients with delayed time from exposure to treatment may benefit from antitoxin therapy in addition to antibiotics^20^, but the guidance is qualitative and does not provide specific quantitative guidance regarding the time window for effective treatment. In light of best evidence suggesting a role for multimodal therapy with both antitoxin and antibiotics together with the reality that most antitoxins are likely to come from public health stockpiled sources, it is crucial to define the critical period from anthrax exposure to successful treatment in order to better inform public health antitoxin distribution goals and responding clinicians’ treatment strategies.

For toxin-mediated diseases, toxin kinetics and antitoxin pharmacokinetics are crucial to guiding selection of appropriate anti-toxin doses and treatment timing. In animal models of inhalational anthrax, PA kinetics are related to disease progression^21^^,^^22^^,^^23^. Current understanding of inhalational anthrax pathogenesis and antitoxin benefits suggest that raxibacumab present in equimolar or higher amounts than PA should be protective^24^. PA kinetics together with raxibacumab pharmacokinetics (PK) can provide an estimate of an effective period for anti-toxin treatment of inhalational anthrax in humans. Raxibacumab PK in healthy human subjects have been reported^25^, and while PA kinetics are known for well-established animal inhalational anthrax models^12^^,^^21^, no such data are available for humans.

By extrapolating human PA kinetics from those in animals, this study sought to determine effective treatment intervals for antibiotics alone or antibiotics with raxibacumab for antibiotic-sensitive inhalational anthrax infections and for raxibacumab alone for antibiotic-resistant events.

## Methods

**Extrapolation of human PA kinetics.** Animal data were compiled from previously completed studies (see Appendix 1, Table A1) conducted in New Zealand White rabbits (*Oryctolagus cuniculus*, 219 rabbits in 5 studies) and cynomolgus macaques (*Macaca fascicularis*, 32 monkeys in 3 studies) challenged with *B. anthracis* spores^12^^,^^17^^,^^21^^,^^23^^,^^24^^,^^26^^,^^27^. The data included individual animal’s PA kinetic parameter values, body weights, and disease characteristics (spore challenge duration, and spore challenge size, therapy and time to therapy, time to first bacteremia by culture, survival status, and survival time).

The previously described PA kinetic parameters^21^ included N_0_, the PA concentration at time 0; λ, the lag time for the first phase of the PA concentration-time profile; μ_m_, the maximum specific growth rate for the first phase of the profile; A, the magnitude of the plateau phase of the profile, expressed as natural log of the ratio of the PA concentration in the asymptotic phase to N_0_; λ_2_, the lag time for the second growth phase in the profile; μ_m,2_, the maximum specific growth rate for the second phase of the profile; and, k_elm,PA_, the first-order PA elimination rate constant. In untreated animals, PA levels follow a rise-plateau-rise pattern, and increase until death occurs. In contrast, in antibiotic-treated animals, antibiotic administration leads to eradication of bacteria, which eliminates PA production so that PA levels decrease.

Potential relationships between PA kinetic parameters and body weight or disease characteristics were assessed by graphical and regression analysis (see Appendix 1, Table A2 and Figure A1). Predictive relationships were selected using visual inspection and an arbitrarily selected correlation coefficient cutoff of ≥0.4. If no suitable predictive relationship for a PA kinetic parameter was identified, the human value was extrapolated as the value from monkeys, since monkeys and humans are both primates and the disease courses are most similar. The exception was k_elm,PA_, since that parameter is only known for rabbits. The range for a PA kinetic parameter in humans was estimated using the predictive relationship between that parameter and a predictor from animals, and substituting the range of the independent variable in humans.

**Simulations of therapeutic intervention.** All simulations were performed using the NONMEM software, Version 7.2.0^28^. Graphical and regression analyses were performed using the R software, Version 2.15.2^29^. The results of all simulations are summarized as median with a 90% prediction interval (PI).

Raxibacumab concentration-time profiles for infected humans were simulated using an existing population PK model and parameters for healthy human subjects, which represented a cross section of the adult US population^25^, with the exception that clearance (CL) was increased by 170% to match the CL difference that was observed between healthy animals and animals with anthrax disease^27^. The profiles were simulated for the recommended 40 mg/kg IV adult dose.

Extrapolated human PA kinetic parameters were used to derive simulated plasma PA concentration-time profiles for the same population as that used for the raxibacumab PK simulations. Inter-individual and residual variability for PA kinetics were set equal to that observed for a large rabbit population^21^. PA profiles were simulated with and without antibiotic-induced PA clearance, to represent profiles for antibiotic-sensitive and antibiotic-resistant B. anthracis infections, respectively. Treatment times ranging from 0 to 14 days after spore exposure were assessed.

**Assessment of protection due to therapeutic intervention.** To assess protection from PA pathogenesis it was presumed that antibiotic killing of bacteria eliminates PA production, allowing net PA clearance, as was observed in rabbit and monkey studies^12^^,^^21^. For raxibacumab, protection was defined as plasma raxibacumab concentrations equimolar to or greater than the concurrent plasma PA concentrations. An assumption of PA clearance after raxibacumab monotherapy (e.g. antimicrobial-resistant anthrax) is plausible since in rabbits and monkeys treated with raxibacumab monotherapy after demonstrated toxemia/bacteremia, some animals survived without subsequent bacteremia or toxemia^12^.

## Results

The extrapolations of the human PA kinetic model parameters are summarized in the Appendix. The data are available at DOI 10.6084/m9.figshare.4763581.

Figure 1 illustrates the anticipated pharmacokinetics of a 40 mg/kg raxibacumab infusion administered over 2 hours. Peak levels are achieved immediately and antitoxin remains in circulation for weeks.

**Predicted median (90% prediction interval) serum raxibacumab concentration-time profile in humans exposed to anthrax spores, following a 40 mg/kg raxibacumab intravenous 2 h infusion dose.**
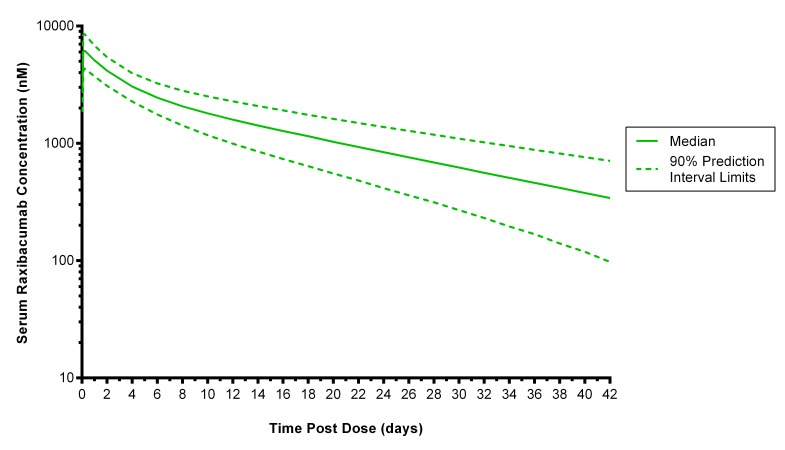
The profile is based on 200 replicate simulations using observed raxibacumab pharmacokinetics in healthy subjects. The exception was raxibacumab clearance, which was increased by the same proportion as clearance was observed to increase between healthy and anthrax infected rabbits or monkeys. The median line represents the central tendency of the results. The 90% PI provides a representation of the expected variability among subjects, with 5% of the subjects falling above and 5% falling below those bounds.

For Figures 2A-2F, the raxibacumab concentration-time profiles are the same as in Figure 1, but vary along the X axis based on when the antitoxin is administered with antibiotics post spore exposure. When raxibacumab is administered together with antibiotics one day after anthrax spore exposure, raxibacumab levels far exceed PA levels for all subjects (Figure 2A). The amount of raxibacumab continues to exceed PA for virtually all simulated patients when administered at increasing durations after exposure up to 6 days (Figures 2B-2D. Figure 2E reveals that a majority (>80%) of subjects remain protected if treatment is initiated by 7 days post exposure. Thereafter, the proportion of subjects protected decreases, with less than one-half of subjects having adequate antitoxin protection if treatment is delayed until 9 days after spore exposure. Table 1 summarizes the percentage of subjects protected for different intervention times.

Figures 2A-2C also shows that antibiotic treatment within 4 days post exposure can be inferred to prevent the onset of exponentially increasing PA levels. This is because a single dose of antibiotic is known to sterilize bacteremia^3^^,^^4^^,^^12^^,^^16^^,^^17^^,^^27^, and hence, eliminates the source of PA production and subsequent exponentially increasing PA levels, and hence. eliminates the source of PA production and subsequent exponentially increasing PA levels.

**Predicted median (90% prediction interval) serum PA and raxibacumab concentration-time profiles in humans exposed to anthrax spores, following concurrent administration of antimicrobial and a 40 mg/kg raxibacumab intravenous 2 h infusion dose.**
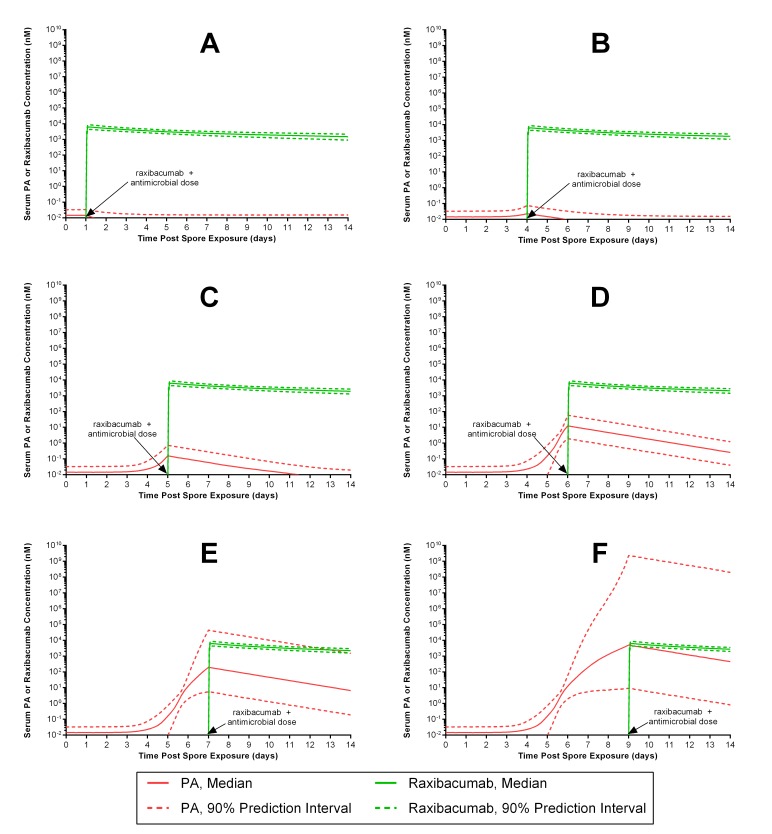
The panels represent simulations for different treatment intervention times: at 1 (A), 4 (B), 5 (C), 6 (D), 7 (E) and 9 (F) days post anthrax spore exposure. Serum raxibacumab levels equimolar to or greater than concurrent PA levels are considered protective. Panels A through D illustrate nearly all subjects have protective raxibacumab levels (lower 90% prediction interval bound for raxibacumab exceeds the upper 90% prediction interval for PA), while panels E and F illustrate that some subjects would not be protected against PA (PA levels can be greater than concurrent raxibacumab levels).

**Figure:**
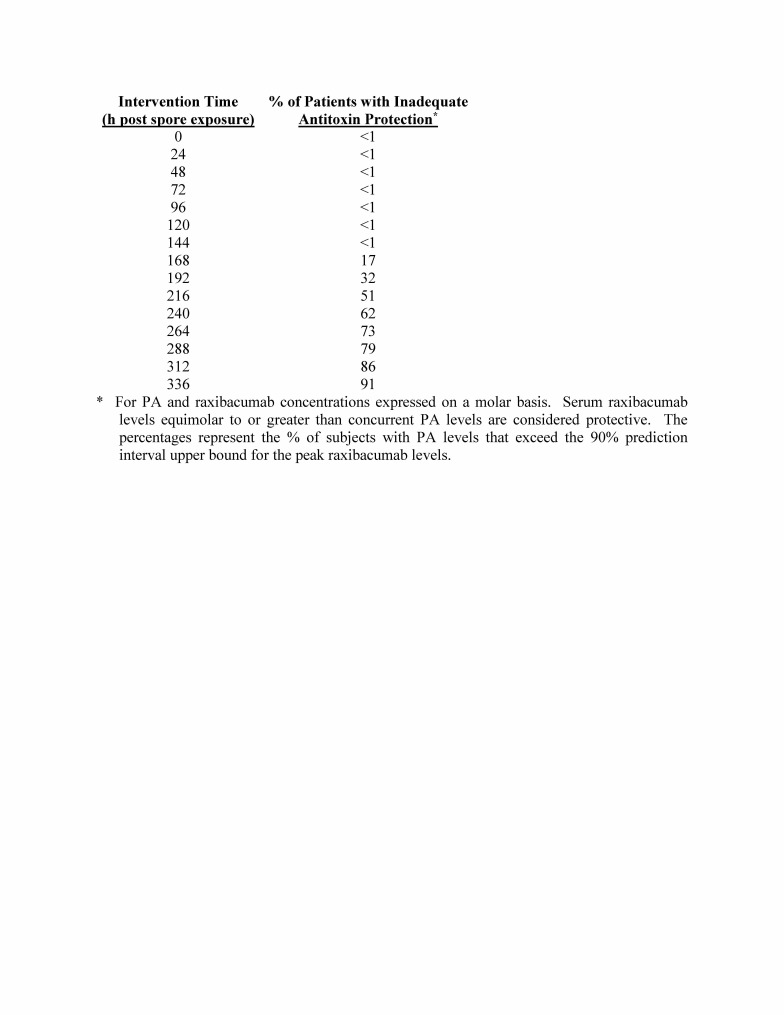
**Table 1:** Summary of predicted anti-toxin protection for co-administered antibiotic and raxibacumab at various times post B. anthracis spore exposure in humans

Figures 3A-3F shows the results of raxibacumab monotherapy for treatment of antibiotic-resistant *B. anthracis* infection (or other situations where antimicrobials cannot be used), with treatment initiated at different times post spore exposure. If treatment with raxibacumab occurs within 6 days from spore exposure, subjects would have protective antitoxin levels (lower 90% PI bound for raxibacumab exceeds the concurrent upper 90% PI bound for PA) (Figures 3A-3D). For treatment that is initiated at later times, ~60% of subjects would not being adequately protected against PA at 7 days and nearly 50% would lack adequate protection at 9 days (Figures 3E and 3F).

**Predicted median (90% prediction interval) serum PA and raxibacumab concentration-time profiles in humans exposed to anthrax spores, following administration of a 40 mg/kg raxibacumab intravenous 2 h infusion dose as monotherapy (e.g., when antimicrobials would not be used, such as for treatment of an antibiotic-resistant B. anthracis strain).**
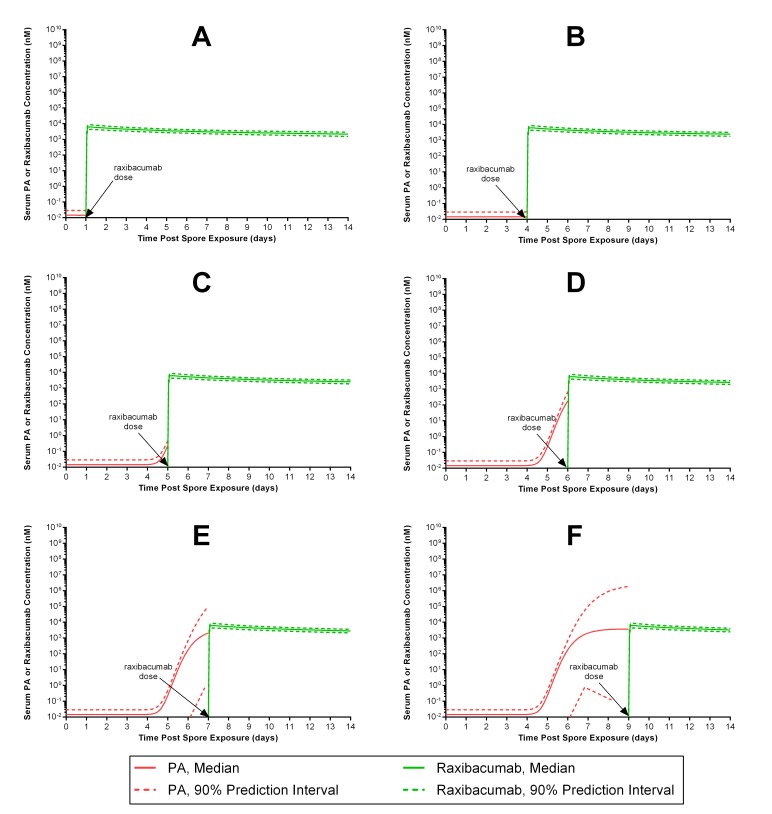
The panels represent simulations for different treatment intervention times: at 1 (A), 4 (B), 5 (C), 6 (D), 7 (E) and 9 (F) days post anthrax spore exposure. Serum raxibacumab levels equimolar to or greater than concurrent PA levels are considered protective. Panels A through D illustrate nearly all subjects have protective raxibacumab levels at some times (lower 90% prediction interval bound for raxibacumab exceeds the upper 90% prediction interval bound for PA), while panels E and F illustrate that some subjects would have inadequate protection (PA levels can be greater than concurrent raxibacumab levels). Please see text for comments re: limitations and assumptions of these simulations.

## Discussion

*B. anthracis* remains a bioterrorism threat, with the potential for thousands or tens of thousands to be exposed after spore release in a densely populated setting^5^^,^^6^^,^^30^^,^^31^. Such numbers of casualties would likely strain and possibly overwhelm the public health and medical care system and it is highly plausible that some patients may not receive immediate prophylaxis or treatment when symptoms are just beginning to manifest. With large numbers of patients presenting to emergency departments seeking care, it is crucial that emergency physicians, intensivists and other clinicians understand the importance of therapeutic options and timing available to them. For patients with inhalational anthrax who progress to severe disease, morbidity and mortality are largely due to the detrimental effects of toxemia, so the timing for effective treatment of toxin-mediated disease must be understood in order to effectively plan for and respond to such a public health emergency. The United States Strategic National Stockpile contains anthrax antitoxins^18^^,^^19^. In order for the antitoxin to be administered, the intentional event must be recognized and the antitoxin distributed to the impacted state(s) and then further distributed to designated health care sites (assuming more than one site will be involved in the treatment of suspected cases). Despite the many consummate professionals who work to optimize these logistics, the simple fact is distribution is likely to take hours to days. Our study provides data-extrapolated guidance to assist development of distribution metrics to ensure that persons in need get timely, effective treatment.

Our simulations provide the first rigorously predicted estimates of the effective treatment period for recommended inhalational anthrax treatment strategies including those involving antitoxins. Administration of raxibacumab concurrent with the first dose of antibiotic is predicted to be beneficial if treatment is initiated within 7 days post spore exposure for the vast majority of subjects ( ≥80%). Our simulations also predict that after 7 days an increasing number of subjects, and after 9 days the majority of exposed individuals, would not have adequate toxin neutralization and may succumb to severe illness. Hence, these simulations support the recommended paradigm of treating as soon as possible after exposure^18^^,^^19^. Importantly, the suggested range from our study may prove difficult but is possible to achieve.

In the 2001 anthrax attack, subjects treated for inhalational anthrax were administered at least 1 antibiotic, and had their bacteremia eradicated within 24 h after antibiotic administration^3^^,^^4^. Similarly, in animal models of inhalational anthrax, bacteremia is generally eradicated within 24 h after antibiotic treatment^16^^,^^17^^,^^27^. When human disease is simulated with the PA elimination expected after antibiotic administration (Figure 2), antibiotic treatment within 4 days post exposure can be inferred to prevent the onset of exponentially increasing PA levels. This suggests that antibiotics alone, if initiated soon enough after *B. anthracis* spore exposure, should be sufficient to protect patients. In contrast, antibiotic intervention at ≥4 days after spore exposure occurs when PA levels are exponentially increasing, and anti-toxin co-administration could still protect the patient from the deleterious toxin effects.

In the United States, raxibacumab is indicated for the prophylaxis of inhalational anthrax when alternative therapies are not available or are not appropriate^13^; for example, exposure to spores of an antibiotic-resistant strain of *B. anthracis*. Our simulations of raxibacumab monotherapy for treatment of antibiotic-resistant *B. anthracis* infection suggest benefit for up to 6 days post-exposure. In rigorously performed rabbit and monkey models, early anti-toxin intervention appears to neutralize toxin and protect the host’s immune cells. The immune cells involved with spore processing are usually rendered dysfunctional by toxin-mediated processes, but toxin neutralization may protect the remaining immune cells and if performed early enough in disease, allows for immune control of the infection. Such an effect would be consistent with the high survival rates observed for raxibacumab monotherapy before the onset of severe symptoms in rabbit and monkey studies^12^. Hence, the 6 day window suggested by this study may allow for immune clearance of infection in humans. In addition, by reducing PA, raxibacumab may offer additional time from disease progression to administer alternate antimicrobials once antibiotic susceptibilities are known and if clinicians believe additional antibiotic therapy to be warranted for some patients.

Although a patient treated at a given time might attain complete toxin neutralization, this alone may not be sufficient to ensure the patient’s survival. Morbidity will be dependent on accrued tissue/organ damage from un-neutralized toxin prior to treatment. So in spite of effective neutralization, organ supportive care may still be needed for the patient to recover.

This simulation assumed administration of raxibacumab’s licensed adult dosing regimen. If faced with a mass casualty event and a limited supply of anti-toxin, administering a reduced dose of anti-toxin might be considered, so that additional individuals could receive anti-toxin. Reducing the dose of anti-toxin would necessarily reduce the magnitude of plasma anti-toxin levels, whereas PA levels would remain unchanged. As a result, using a lower anti-toxin dose would increase the proportion of patients with anti-toxin levels inadequate for toxin neutralization and at risk for deleterious toxin effects and increased morbidity. Hence, anti-toxin dose reduction likely puts more patients at risk.

This work’s limitations relate to the assumptions used in extrapolation of the human PA kinetic parameters and to the lack of human data related to the predicted outcomes. The first limitation is mitigated by the observation that the extrapolated human PA kinetics are consistent with disease progression in humans while preserving the differences in disease progression between animals and humans. The PA profiles across species have similar shapes, but reflect the differences in timing of disease events across species. That is, PA levels rise soonest, reach plateau earliest, and enter the terminal increasing phase earliest in rabbits, the species with the most rapid progression of inhalational anthrax. (Supplement Figure 1). In contrast, the timeline of PA increase was the most delayed for the human model; reassuringly humans have the most protracted course of disease, even when compared to monkeys. Although these extrapolations and simulations provide a leap forward for guidance for treatment decisions, actual measured PA kinetics for humans would supersede the extrapolated human PA kinetics in this report.

The limitations caused by the lack of human data manifest in several ways. The lack of measured PA exposure in humans prevents confirmation of the simulations with observed data, including the effect of antimicrobial therapy on PA levels. Also, raxibacumab PK have only been measured in healthy subjects, not anthrax-infected subjects. Since it is known that raxibacumab CL was increased in anthrax-infected animals relative to healthy animals, we applied a similar assumption to simulate human raxibacumab profiles with increased CL. Importantly this assumption only accounts for the animal model of disease and does not reflect the potential impact of supportive care (e.g. fluid resuscitation) on PA kinetics and raxibacumab PK. While both may be impacted in a similar magnitude and not significantly change the estimated time period, the true impact of human critical illness and supportive care on the time window for neutralization is nearly impossible to convincingly predict. The lack of confirmatory data on raxibacumab PK and PA effects in anthrax-infected humans will remain unless natural or intentional cases of inhalational anthrax occur.

In summary, extrapolating human PA kinetics from animal models provided a basis for simulations evaluating therapeutic interventions for human inhalational anthrax. Our study suggests that intervention with antibiotic alone within 4 days after spore exposure is sufficient for survival for nearly all persons, and that use of anti-toxin together with antimicrobials extends this treatment window if administered within the first week. At later intervention times, the combination of antimicrobials with anti-toxin would not provide complete protection for all subjects. When it is not possible to utilize antimicrobials in combination with anti-toxin (e.g., an antibiotic-resistant strain), anti-toxin monotherapy within 6 days post spore exposure should neutralize toxin and promote survival of the patient’s immune cells, allowing their immune system to prevent an infection from becoming established. These recommendations will need to be re-evaluated if additional human PA kinetic data and raxibacumab PK data in critically ill patients become available.

## Corresponding Author

Alfred Corey, ACPharmaco, LLC, 1043 Heather Lane, Wake Forest, NC 27587 email: acpharmaco@gmail.com

## Competing Interests

Dr. Rubinson has received a one-time financial honoraria in early 2015 for participating on a scientific advisory board for GlaxoSmithKline related to raxibacumab. This has been his only relationship with the company. All work on this manuscript was undertaken without any financial renumeration. The conceptualization, analyses and conclusions of the manuscript were all derived without any influence or direct discussion with GlaxoSmithKline.

Dr. Hanfling serves as an intermittent consultant to GlaxoSmithKline on matters related to anthrax diagnosis, management, treatment and modeling, and served as the Chairman of a Scientific Advisory Board convened by the company in February 2015 to discuss the added benefits of raxibacumab in the setting of a mass casualty event. All work on this manuscript was undertaken without any financial renumeration. The conceptualization, analyses and conclusions of the manuscript were all derived without any influence or direct discussion with GlaxoSmithKline.

Mr. Corey was employed by GlaxoSmithKline (GSK), the manufacturer of raxibacumab, at the time most of the analyses were performed. The conceptualization, analyses and conclusions of the manuscript were all derived without any influence or direct discussion with GlaxoSmithKline.

## Data Availability

The data have been placed on http://figshare.com/, with a DOI of 10.6084/m9.figshare.4763581.

The extrapolations of the human PA kinetic model parameters are summarized in the Appendix. The data are available at DOI 10.6084/m9.figshare.4763581.

## Appendix

**Animal studies used as data sources.** Data were compiled from various studies (Table A1) conducted in New Zealand White rabbits (*Oryctolagus cuniculus*, 219 rabbits in 5 studies) and cynomolgus macaques (*Macaca fascicularis*, 32 monkeys in 3 studies) challenged with *B. anthracis* spores^12^^,^^17^^,^^21^^,^^23^^,^^24^^,^^26^^,^^27^. The data included individual animal’s PA kinetic parameter values, body weights, and disease characteristics (spore challenge duration, and spore challenge size, therapy and time to therapy, time to first bacteremia by culture, survival status, and survival time.

**Figure:**
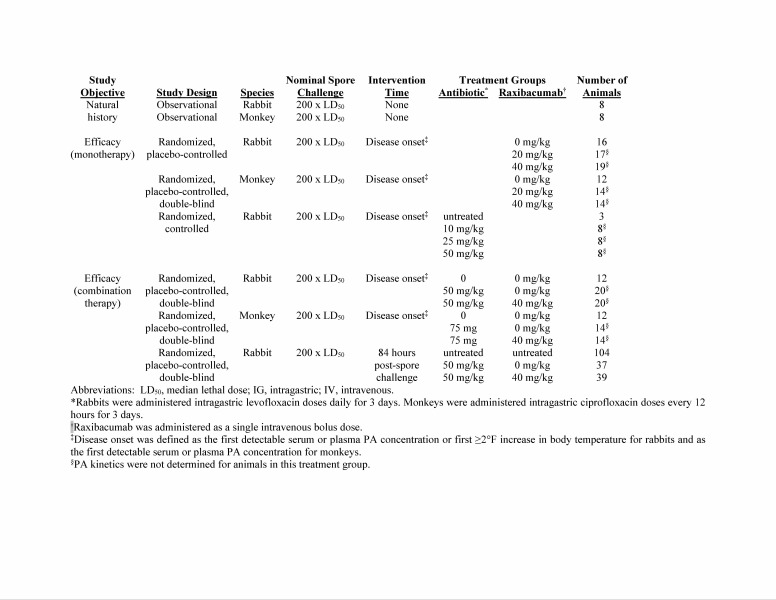
**Table A1:** Description of rabbit and monkey studies used as data sources

**Protective antigen kinetics.** The mathematical models used to describe protective antigen (PA) kinetics are described in reference 21.

**Supplemental results - extrapolation of human PA kinetics.** Human PA kinetics were extrapolated from relationships between animal PA kinetic parameters and animal characteristics. The assessments of possible relationships between PA kinetics and animal characteristics are summarized in Table A2. The relationships selected for use in extrapolation of human PA kinetic parameters are illustrated in Figure A1. Time to 1st detected bacteremia was a predictor for λ, and survival time was a predictor for μ_m_, A, and λ_2_. Table A3 provides the extrapolated human PA kinetics, and for comparison also includes PA kinetics observed in rabbits and monkeys. Figure A2 illustrates the predicted human PA concentration-time profile in the absence of any intervention and includes corresponding profiles in rabbits and monkeys for comparison.

**Figure:**
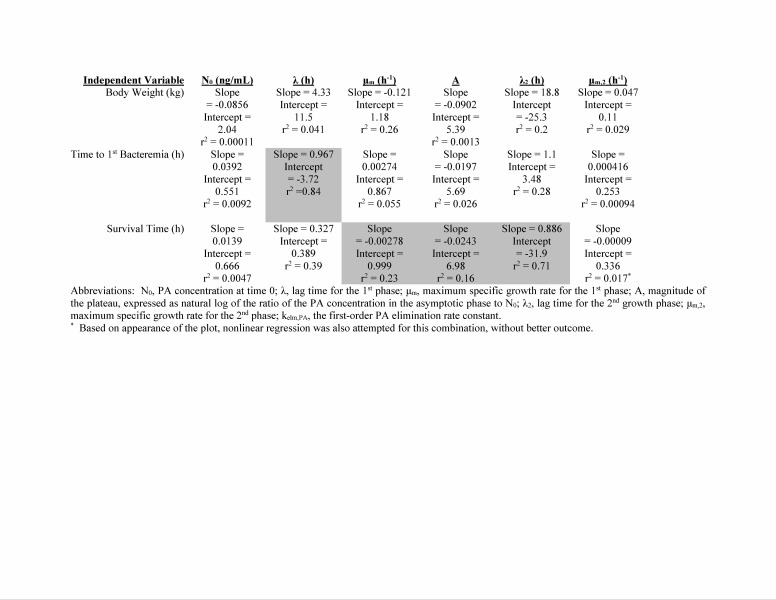
**Table A2:** Summary of assessments for potential relationships between PA kinetics and animal charac

**Figure:**
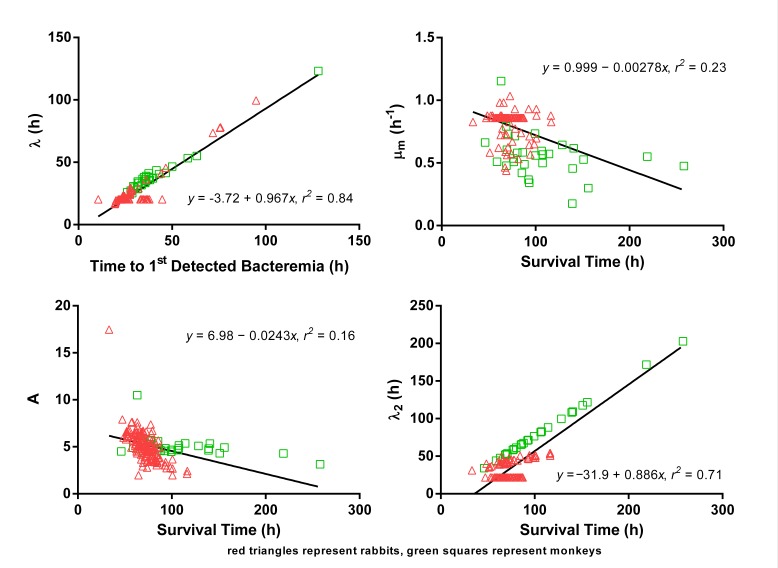
**Fig. A1:** Relationships of rabbit and monkey PA kinetics to disease events that were used for extrapolation of human PA kinetics

**Figure:**
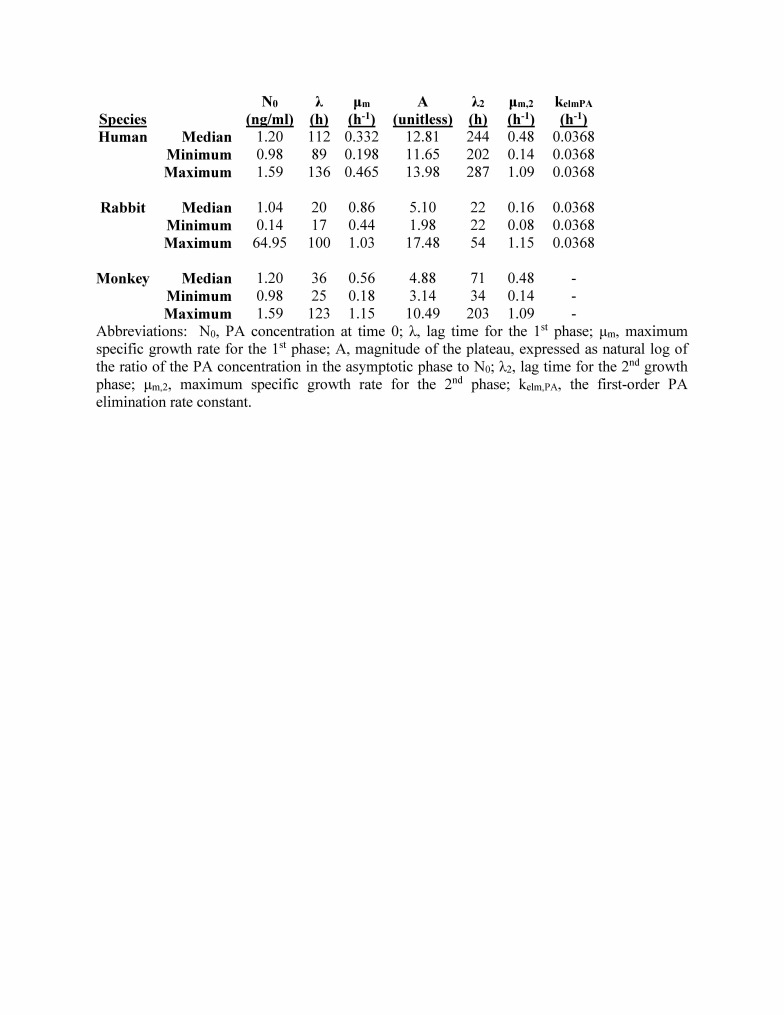
**Table A3:** Extrapolated human PA kine

**Figure:**
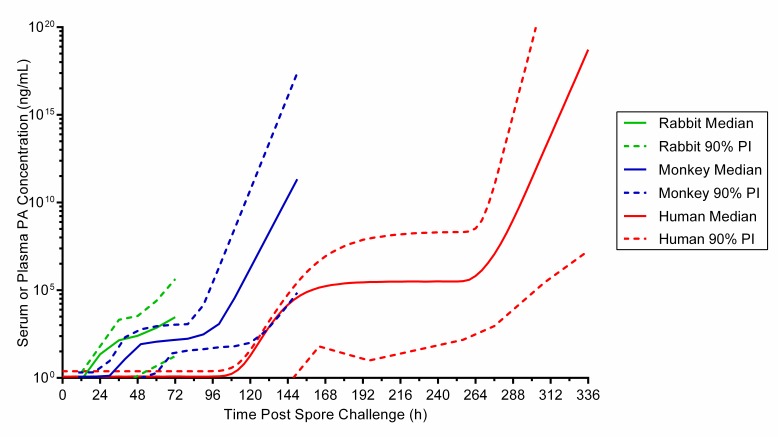
**Fig. A2:** Predicted PA profile in humans versus observed PA profiles in rabbits and monkeys after an inhaled B. anthracis spore challenge, without treatment interv
